# Identification of QTLs Linked to Phenological and Morphological Traits in RILs Population of Horsegram (*Macrotyloma uniflorum*)

**DOI:** 10.3389/fgene.2021.762604

**Published:** 2022-01-25

**Authors:** Megha Katoch, Rushikesh Sanjay Mane, Rakesh Kumar Chahota

**Affiliations:** Department of Agricultural Biotechnology, College of Agriculture, CSK HP Krishi Vishvavidyalaya, Himachal Pradesh, India

**Keywords:** *Macrotyloma uniflorum*, genetic linkage map, QTL map, phenotypic variation, phenological traits, morphological traits

## Abstract

Horsegram [*Macrotyloma uniflorum* (Lam.) Verdc.] is an important legume but understudied in terms of its genetic improvement. Genetic information on various phenological and morphological traits may help in the utilization of new genes for breeding in horsegram and thus affect agronomic practices and crop yield. A total of 162 recombinant inbred lines derived from intraspecific crosses between HPKM249 × HPK4 was used to construct a genetic linkage map and to identify quantitative trait loci (QTLs) associated with phenological and morphological traits. Of the total 2011 molecular markers, which were screened on parental lines for polymorphism survey, 493 markers were found to be polymorphic and used for genotyping of recombinant inbred line population. Out of 493 polymorphic markers, 295 were mapped on ten linkage groups at LOD 3.5 spanning a total distance of 1,541.7 cM with an average distance between markers of 5.20 cM. Phenotypic data of two years at two different locations were used to identify QTLs by composite interval mapping A total of four QTLs (LOD ≥2.5) for phenological traits (days to 50% flowering, reproductive period and days to maturity) and seven QTLs (LOD ≥2.5) for morphological traits (plant height, primary branches and secondary branches) were detected across different environments. The phenotypic variation explained by QTLs ranged from 6.36 to 47.53%. The present study will help to augment scanty genomic information in this orphan crop that would provide genomics tools to breeders for its genetic enhancement through molecular-assisted selection.

## Introduction

*Macrotyloma uniflorum* (Lam.) Verdc (commonly called Horsegram) is an important legume and fodder crop of Asia and Africa, where it is grown as a staple food crop from prehistoric times. It has diploid chromosome number 2n = 20 (Cook et al., 2005) with a genome size of 398 Mbps (Shirasawa et al., 2021). The genus *Macrotyloma* comprises 32 wild species distributed in African, Australian, and Indian subcontinents. Amongst them, *Macrotyloma uniflorum* var. *uniflorum* is regarded as the only cultivated species grown in the Indian subcontinent andconsidered to be originated in Southern India (Vavilov, 1951; Zohary, 1970).

Horsegram is cultivated as food legume in India, Sri Lanka, Mauritius, Nepal, Malaysia, and Myanmar by the poor people of the marginal areas, whereas in Africa and Australia it is mainly cultivated for animal feed (Asha et al., 2006). Owing to its valuable medicinal properties mentioned in Ayurveda, it is cultivated on a larger areas in India as compared to other countries. In India, Andhra Pradesh, Karnataka, and Tamil Nadu are the major horsegram producing states and cover approximately an area of 0.31 million ha and the collective production is 0.13 million tonnes with a yield of 430 kg/ha (DES 2016–2017).

The ever-increasing global population accompanied by degradation in cultivated land has put precocious pressure on breeders to enhance food grain production of non conventional crops so that food can be provided for everyone. Of the several hundred plant species known, only 120 species are cultivated for human food. However, of these, only nine crops supply approximately 75% of global plant-derived energy, wheat, rice, and maize being the top three crops (FAOSTAT 2021). Therefore, there is a dire need to explore other plant species/crops that bears the capacity to meet the increasing food supply-demand with simultaneous better nutritional values. Horsegram is one of the underprivileged crop and exhibit immense potential to cope with the increasing demand of food. Itpossesses inherent capability to grow under drought-like situations (Reddy et al., 1990), grow in varied temperature conditions (Ramya et al., 2013), tolerant to heavy metal stress (Sudhakar et al., 1992), and having a high percentage of protein, antioxidants, fiber and several important vitamins like Vitamin A, Vitamin B1, Vitamin B2, Vitamin B3 and vitamin C (Sodani et al., 2004; Reddy et al., 2005). Additionally, it has nitrogen fixation capacity which aids in improving the fertility of the soil. Also, the description of horsegram in Ayurveda is known for centuries and is widely used in the treatment of urinary stones and urinary diseases, regulates the abnormal menstrual cycle in women and is used to treat high fever, throat infection, cough, hiccups, and worms (Yadava and Vyas, 1994; Chunekar and Pandey, 1998; Neelam, 2007; Ravishankar and Vishnupriya, 2012). Its valuable nutritional and medicinal properties make it a crop of interest and potential food source of the future (National Academy of Sciences 1978).

The major bottleneck in horsegram productivity is the low genetic potential of most of the released varieties, which are generally developed from a narrow genetic base. Attempts should have been made to genetically improve such crops by combining favorable QTLs for various target traits in a single plant genotype (Wu, 1998). Lack of genetic variation and genomic information on important plant traits is a major obstruction to initiate a systematic breeding program in horsegram. The scarcity of genomic resources challenged/prompted us to undertake Simple Sequence Repeat (SSR) marker developed in the related well-characterized legume species. We initiated the horsegram marker development program in the year 2012 and currently, we have a repertoire of more than 10,000 SSRs identified from well-characterized legumes including genomic and genic SSRs developed from horsegram transcriptome and genomic sequences. These genomic resources can be assessed in horsegram database (www.hillagric.ac.in:1005) and now being employed to improve grain yield and other agronomic traits. Phenological and morphological traits are important traits and knowledge of their genetics may help in the utilization of new genes for breeding and thus affect agronomic practices and crop yield. However, these traits have a complex phenotype, polygenic in nature, and are quantitatively inherited. Hence, mapping quantitative trait loci (QTLs) associated with genomic regions harboring genes for these traits represent a promising strategy for undertaking marker-aided breeding and trait improvement. Till now no such study has been reported in horsegram and thus needs to be determined. Therefore, for the genetic improvement of horsegram construction of fine linkage map and identifying QTLs linked to various important agronomic traits is essential which will increase the genomic resources and knowledge of genetics of these traits.

In the present study, we report the development of a linkage map generated with 295 molecular markers and quantitative trait loci (QTL) mapping of phenological and morphological traits in a recombinant inbred line (RIL) population derived from the intraspecific cross between HPKM249 × HPK4. The information presented in the study will help to dissect morphological and phonological traits with the help of molecular markers and provide breeders with genomics tools to select desirable plant type.

## Materials and Methods

### Mapping Population

**FIGURE 1:**
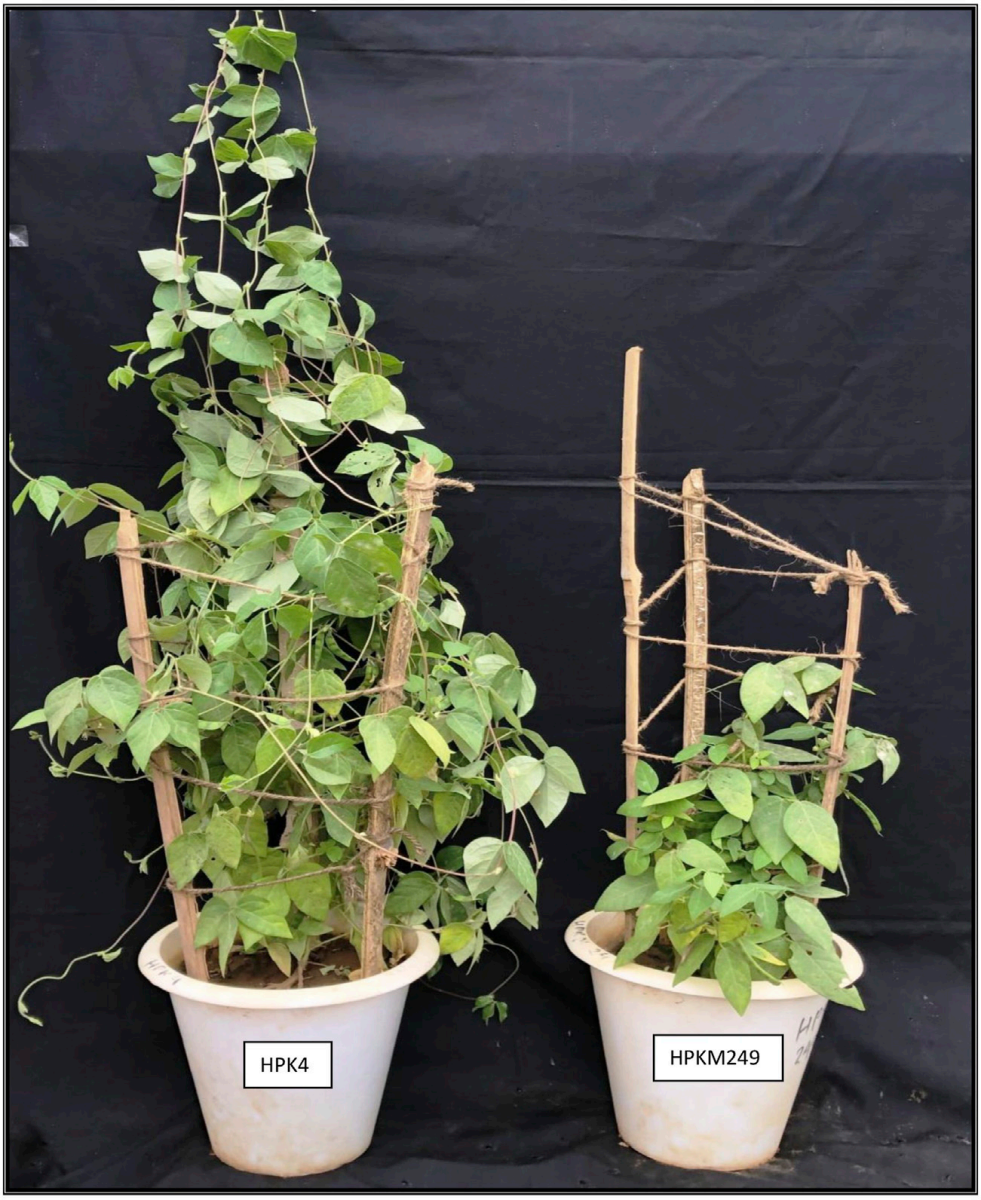
Morphology of the two contrasting parents.

Mapping population consisting of 162 RILs derived from an intraspecific cross of HPKM249 × HPK4 was evaluated for morphological and phonological traits. The mapping population was developed through Single Seed Descent (SSD) method from F_2_ to F_8_ generations. The parental lines (HPKM249 & HPK4) show contrasting characteristics to each other for the traits under study (Figure 1 and Table 1).

**TABLE 1:** Variations in parents for different traits.

Trait	HPK4	HPKM249
Days to flowering	60–65	30
Days to maturity	120–124	80–82
Plant height (cm)	100.0	35.0–40.0
Growth type	Indeterminate	Determinate
Growth habit	Twining	Bush type
Maturity type	Asynchronous	Synchronous

### Phenotyping

During the crop season, F_8_ progenies were planted in the experimental areas of department of Agriculture Biotechnology, CSK HPKV, Palampur, H.P. (latitude, 32.11 and longitude, 76.53) and at Hill Agricultural Research & Extension Centre, Bajaura, H.P. (latitude, 31.85 and longitude, 77.16) using Augmented Block Design (ABD) with two replications and four checks, VLG-1, HPKM249, HPK4 and HPK317 repeated after 20 rows. Each 1-m line consisted of 10 plants spaced 30 cms apart. Standard agronomic practices were followed for raising healthy crop. Phenotyping of various phenological and morphological traits were carried out at these locations (Palampur and Bajaura) over a period of two seasons (2016 and 2017). Data on five randomly taken plants in between the first and last plants of the same line was recorded. Three phenological traits viz. days to 50% flowering (FL), days to maturity (MT) and reproductive period (RP) and three morphological traits viz. plant height (PH, cm), number of primary branches (PB), and number of secondary branches (SB) were recorded. Plant height, number of primary branches, and number of secondary branches were measured just before the physiological maturity of the plant by taking readings on five plants in each line and averaging before analysis. Days from the date of sowing to the date when 50% of the plants in a line showed the first fully open flower and days from sowing to physiological maturity when 90% of the plants had turned brown were recorded for calculating days to 50% flowering (FL) and days to maturity (MT) respectively. The reproductive growth period (RP) was calculated as the days between the start of flowering and physiological maturity.

### Statistical Analysis

Traits distribution was studied using skewness and kurtosis statistics by Past 3.25 software. Pearson correlation coefficients and frequency distribution among different traits were calculated using the same software. The parental phenotypic variance was analyzed using ANOVA.

### Genotyping

Young leaf tissues (0.5–1 g) of 162 RILs individuals along with parents (HPK4 and HPKM249) were used for isolation of genomic DNA using the modified cTAB method (Murray and Thompson, 1980). Concentration (ng/µl) and purity of isolated DNA was checked on agarose gel electrophoresis and quantified on a microvolume spectrophotometer (Biospec-nano, Shimadzu Biotech, United States) using Tris EDTA as blank. PCR-based markers from different sources were used to screen polymorphism between parent HPK4 and HPKM249. A total of 2011 PCR primers consisting of 63 Expressed Sequence Tag Simple Sequence Repeats (EST SSRs), 403 genic Simple Sequence Repeats (gSSRs), 387 genomic Simple Sequence Repeats (geSSRs), 24 drought specific Simple Sequence Repeats (dsSSRs), 300 Simple Sequence Repeats (SSR) from other legumes, 450 Random Amplified Polymorphic DNA (RAPD) markers, and 384 Conserved Ortholog Set (COS) markers of Cook’s Lab UCD, United States were employed in the present study. The polymorphic primer pairs were then used for genotyping of the mapping population.

For amplification using SSR markers, a total reaction mixture of 10.0 µl volume was prepared to contain 4.80 µl of sterilized distilled water, 2.0 µl of template DNA (13 ng/μl), 0.5 µl each of forward and reverse primer (5 µM), 0.5 µl of MgCl2 (25 mM), 1.0 µl of 10X PCR buffer (10 mM Tris-HCl, 50 mM KCl, pH 8.3), 0.5 µl of dNTP mix (0.2 mM each of dATP, dGTP, dCTP and dTTP) and 0.2 µl of *Taq* polymerase (5U/µl). The reaction mixture for RAPD markers consisted of 1.5 of µl DNA, 2.5 µl of 10X PCR buffer (10 mM Tris, pH 9.0, 50 mM KCl, 0.01% Gelatin, 1.5 mM MgCl2), 0.2 µl of dNTPs (25 mM), 1.0 µl of MgCl2 (25 mM), 1.0 µl of primer (2 μM/μl), 0.1 µl of Taq DNA polymerase (5 U/µl) and made final volume of 25 µl by adding deionised water. The PCR amplification of genomic DNA was performed on Veriti 384^®^ Thermal Cycler (Applied Biosystems, CA, United States). The PCR program for amplification was an initial denaturation cycle at 94°C for 5 min, followed by 45 cycles of denaturation at 94°C for 1 min, annealing at 50°C–65°C (for SSRs), and 37°C (for RAPDs) for 1 min and extension at 72°C for 2 min and final extension at 72°C for 7 min. The amplified products were resolved on either 6% Polyacrylamide Gel Electrophoresis in 1X TBE or 3% metaphor agarose gel (Lonza) in 1X TAE buffer depending on the size difference between amplified DNA along with the size markers (100 bp DNA ladder). The fragments were visualized using Gel-Documentation Unit (ENDUROTM GDS Gel Documentation System, United States) or silver-staining procedure.

The input file for linkage map construction was prepared manually by the scoring of amplified bands. HPKM249 type banding pattern was scored as “A”, HPK4 type banding pattern was scored as “B” and heterozygous loci was scored as “H”, whereas for RAPD markers the absence (0) and presence 1) of bands were recorded. Sizes of amplified fragments were noted by using a 100-bp DNA ladder (Fermentas, Lithuania).

### Genetic Linkage Map and QTL Analysis

The genetic linkage map construction was performed with scored genotypic data file using JOINMAP^®^ 4.1 program (van Ooijen, 2006). The LOD threshold >3.0 and <8.0 with a step-up of 0.5 was considered significant for identifying different linkage groups and clustering of markers on them. The linkage groups which show the highest number of markers with maximum linkage among them at different LODs values were selected.

For quantitative trait analysis, QTLs were identified using the composite interval mapping (CIM) method (Zeng, 1993; Zeng, 1994) implemented in Windows QTL Cartographer V2.5 software (Wang et al., 2005). The walking speed selected for QTLs was 2 cM with a window size of 10 cM using the Zmapqtl standard model 6. The forward regression algorithm was used to obtain cofactors. To calculate a genome-wide threshold for LOD score a 1000-permutation test at a significance level of *p* = 0.05 for shuffling genotypes with the phenotype means was performed (Doerge and Churchill, 1996). A LOD threshold score of ≥2.5 was selected for identification of the QTLs on the horsegram LGs. The location of QTLs with 95% confidence intervals was estimated by one LOD interval around the QTL peak (Mangin et al., 1994). The estimated additive effect and the percentage of phenotypic variation explained (based on the R2 value) by each putative QTL was calculated by the Zmapqtl procedure using the software with the CIM model. The QTL map was prepared with MapChart 2.32 software (Voorrips, 2002).

## Results

### Trait Variations in Parents and RIL Population

Horsegram being an orphan crop, very little has been understood about the genetic structure of its traits. We analyzed six phenological and morphological traits as described in Table 2 which represents the descriptive statistics for all the traits. Parental lines, HPKM249 and HPK4 which were used for developing the mapping population found to have significant differences for all the traits studied. Three phenological traits, namely days to 50% flowering (FL), reproductive period (RP), and days to maturity (MT) are important indicators of maturity and were used for phenotyping of the RILs population. Phenotyping of FL showed significant genetic variability for RILs in different years and locations. HPKM249 flowered in 36 days as compared to 54 days of HPK4 during 2016 at Palampur and similar results were observed at Palampur during 2017, whereas at Bajaura during 2017, HPKM249 flowered in 32 days as compared to 57 days of HPK4. The range for days to flowering among RILs varied from 30–58 days in 2016 at Palampur, 32–52 days in 2017 at Palampur, and 31–57 days in 2017 at the Bajaura location. Further, no significant difference was found among RILs in different years and locations. A similar trend was observed for RP and MT with no significant difference among RILs in different years and locations.

**TABLE 2:** Mean performance of parents and RILs for phenological and morphological traits.

Traits	Year	Location	HPKM249	HPK4	Range (RIL)	Mean	Sd
Morphological							
Plant height (PH)	2016	PLP	38.0	101.0	34.0–98.0	68.3	14.0
	2017	PLP	41.0	99.0	48.0–106.0	72.9	12.0
	2017	BJR	39.0	99.0	60.0–145.0	91.7	18.8
	Combined	—	39.8	99.3	52.1–116.8	78.4	13.3
Primary branches (PB)	2016	PLP	6.7	2.6	1.0–6.0	2.5	0.9
	2017	PLP	6.4	2.5	1.6–5.3	3.0	0.7
	2017	BJR	10.0	3.0	2.5–9.5	6.0	1.4
	Combined	—	7.7	2.7	1.9–5.7	3.9	0.7
Secondary branches (SB)	2016	PLP	8.0	5.2	1.8–14.0	5.0	1.5
	2017	PLP	12.2	3.6	3.7–10.7	6.5	1.4
	2017	BJR	14.0	5.0	5.0–18.0	10.8	2.4
	Combined	—	12.1	4.3	5.0–11.0	7.7	1.2
Phenological							
Days to 50% flowering (FL)	2016	PLP	36.0	54.0	30.0–58.0	41.2	5.9
	2017	PLP	36.0	54.3	32.7–52.7	41.6	4.7
	2017	BJR	32.0	57.5	31.0–57.0	40.9	4.9
	Combined	—	34.7	55.3	33.5–52.2	41.3	4.6
Reproductive period (RP)	2016	PLP	39.0	64.0	19.0–77.0	50.4	10.6
	2017	PLP	46.7	62.7	32.3–74.3	52.8	8.7
	2017	BJR	48.0	56.5	37.0–73.5	55.4	7.8
	Combined	—	45.8	60.8	34.7–73.7	53.3	7.8
Days to maturity (MT)	2016	PLP	75.0	118.0	71.0–115.0	91.6	10.4
	2017	PLP	82.7	117.0	71.6–114.0	94.4	9.7
	2017	BJR	80.0	114.0	78.5–112.5	96.3	9.2
	Combined	—	80.5	116.1	74.1–111.0	94.6	9.0

RIL population was phenotyped for various morphological traits like plant height (PH), number of primary branches (PB), and number of secondary branches (SB). Plant height (PH) varied from 34–98 cm and 48–106 cm at Palmapur for the year 2016 & 2017, respectively. A significant difference was observed for PH in 2017 at Bajaura (60–145 cm) as compared to the Palampur location. Similarly, PB varied from one to six branches at Palampur in both years (2016 and 2017), however a significant difference was observed for PB in 2017 at Bajaura, which varied from 3–10. Further, significant differences for PH and PB among RILs were observed in both seasons and locations. A similar result was observed for SB with significant differences among RILs in both years and locations.

**FIGURE 2:**
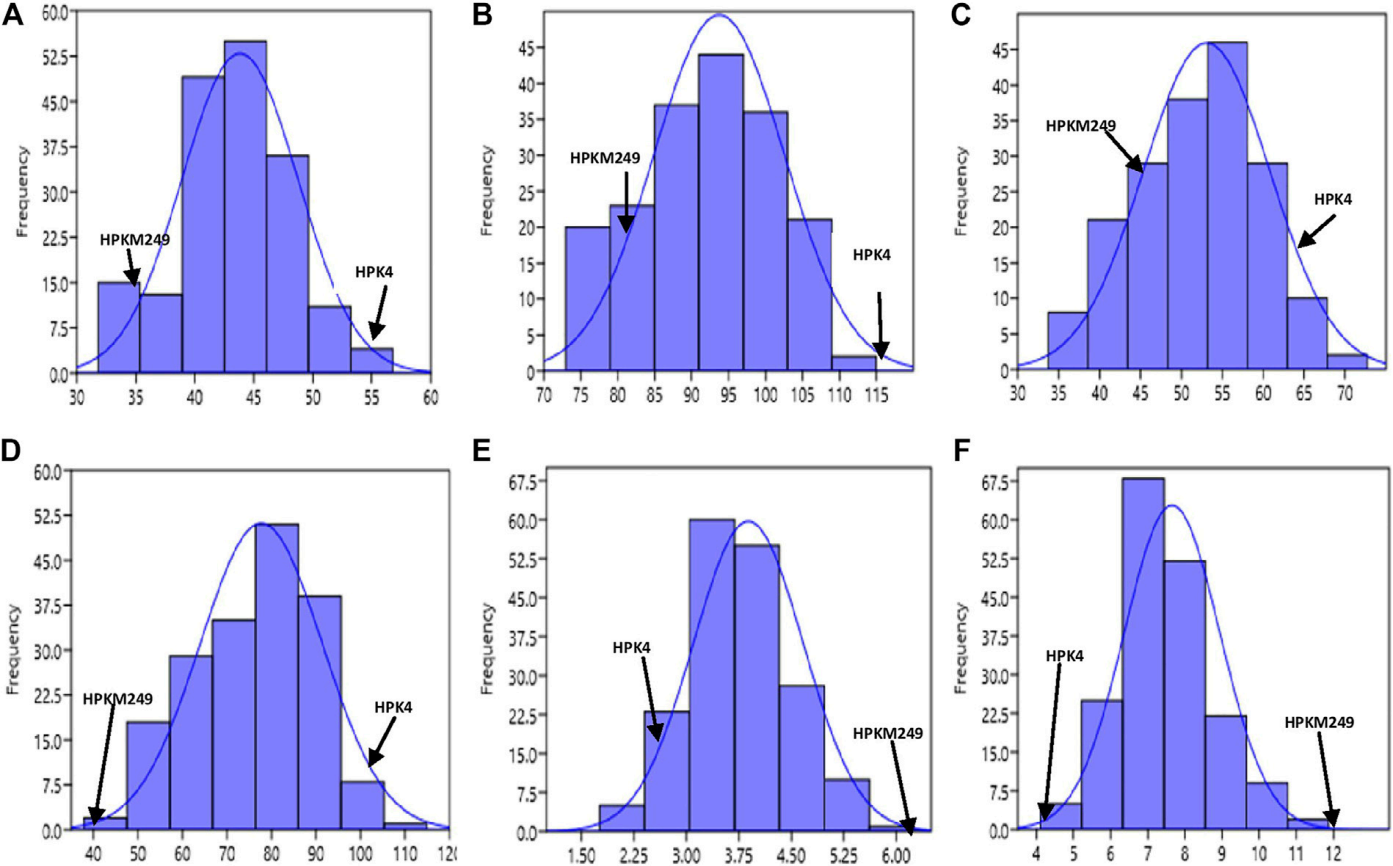
Frequency distribution curve of **(A)** Days to 50% flowering **(B)** Days to maturity **(C)** Reproductive period **(D)** Plant height **(E)** No. of primary branches **(F)** No. of secondary branches.

**FIGURE 3:**
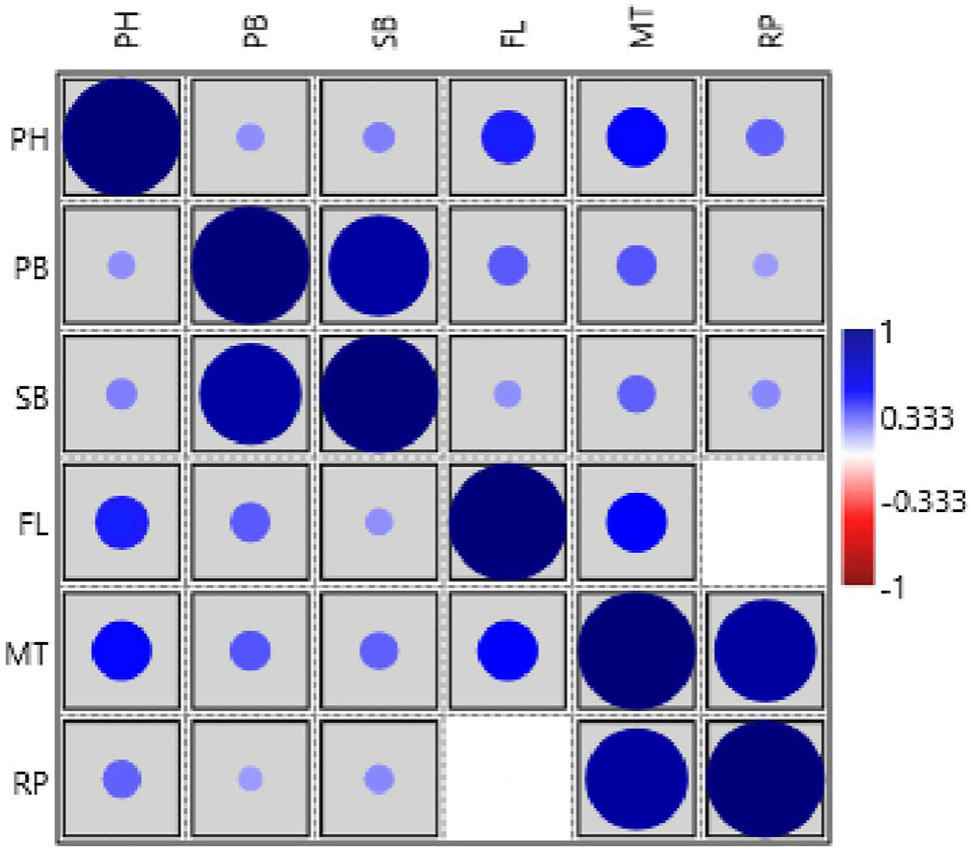
Pearson’s correlation matrix among different traits analyzed in the HPM249 HPK4 RILs (PH-Plant height, PB-No. of primary branches, SB-No. of secondary branches, FL-Days to 50% flowering, MT- Days to maturity, RP- Reproductive period).

The phenotypic values showed a normal frequency distribution, which is a typical characteristic of quantitative traits (Figure 2). Correlations values among traits in RIL populations showed that the traits were positively correlated with each other and were statistically significant (*p* < 0.05). The maximum correlation was found between MT and RP (r = 0.86) followed by PB and SB (r = 0.85) (Figure 3 and Table 3). All traits showed positive correlations among themselves except for FL and RP which showed negative correlation with each other in all environments. The ANOVA of 162 RILs for different locations and environments showed significant variation for all the traits.

**TABLE 3:** Pearson correlation coefficients among traits evaluated in the RIL population.

	PH	PB	SB	FL	MT	RP
Plant Height	1.00	0.22	0.25	0.44	0.49	0.31
No. of Primary branches	0.22	1.00	0.85	0.32	0.33	0.19
No. of Secondary branches	0.25	0.85	1.00	0.22	0.31	0.23
Days to 50% flowering	0.44	0.32	0.22	1.00	0.50	−0.01
Days to maturity	0.49	0.33	0.31	0.50	1.00	0.86
Reproductive period	0.31	0.19	0.23	−0.01	0.86	1.00

### Parental Polymorphism and Genotyping of Mapping Population

The polymorphism survey was done on parental lines using 1177 SSR primer pairs [63 (Horsegram EST SSRs) + 403 (Horsegram genic SSRs) + 387 (Horsegram genomic SSRs) + 24 (drought specific SSRs) + 300 (SSRs from other legumes viz. red clover and *Medicago*)] of which 430 were found to be polymorphic (Table 4). Along with this, 450 RAPD primers were also screened out of which 55 were found to be polymorphic and in addition 384 COS markers of *Medicago truncatula* were screened out of which 8 were found to be polymorphic (Table 4). A total of 2011 primers were screened, of which 493 were found to be polymorphic and were further used for genotyping of 162 individuals (Table 4). The size of amplified bands produced by 493 polymorphic primers ranged between 100 and 250 bp. The genotyping data thus obtained was scored manually and used as the input file for the construction of the horsegram linkage map.

**TABLE 4:** List of molecular markers used for construction of linkage map of horsegram.

S. No	Marker	Markers screened	Polymorphic markers	Percent polymorphism (%)	Markers mapped	Source
1	HUGMS	63	36	57.14	15	EST SSRs (Sharma et al., 2015)
2	MUMS	200	55	27.50	45	Genic SSRs (Sharma et al., 2015)
3	MUMST	100	37	37.0	22	Genic trirepeats (Sharma et al., 2015)
4	MUMSD	103	44	42.72	20	Genic Direpeats (Sharma et al., 2015)
5	MUGSSR	99	42	42.42	31	Genomic SSRs (Chahota et al., 2017)
6	MUSSR	50	24	48.0	16	Genomic SSRs (Chahota et al., 2017)
7	MUGR	94	30	31.91	20	Genomic SSRs (Chahota et al., 2017)
8	MUD	96	28	29.17	13	Genomic SSRs (Kaldate et al., 2017)
9	MUGSR	48	8	16.67	7	Genomic SSRs (Chahota et al., 2017)
10	RAPD	450	55	12.22	22	Operon Tech, United States of America and Fred Muehlbauer, United States of America
11	Drought specific primers	24	5	20.83	4	Charu and Manoj 2011
12	RcSSRs	196	88	44.90	56	Sato et al., 2005
13	MtSSRs	104	33	31.73	17	Eujayl et al., 2004
14	COS	384	8	2.08	7	Douglas R. Cook, UC, Davis, United States of America
—	Total	2011	—	493	24.52	295

### Construction of Genetic Linkage Map

**FIGURE 4:**
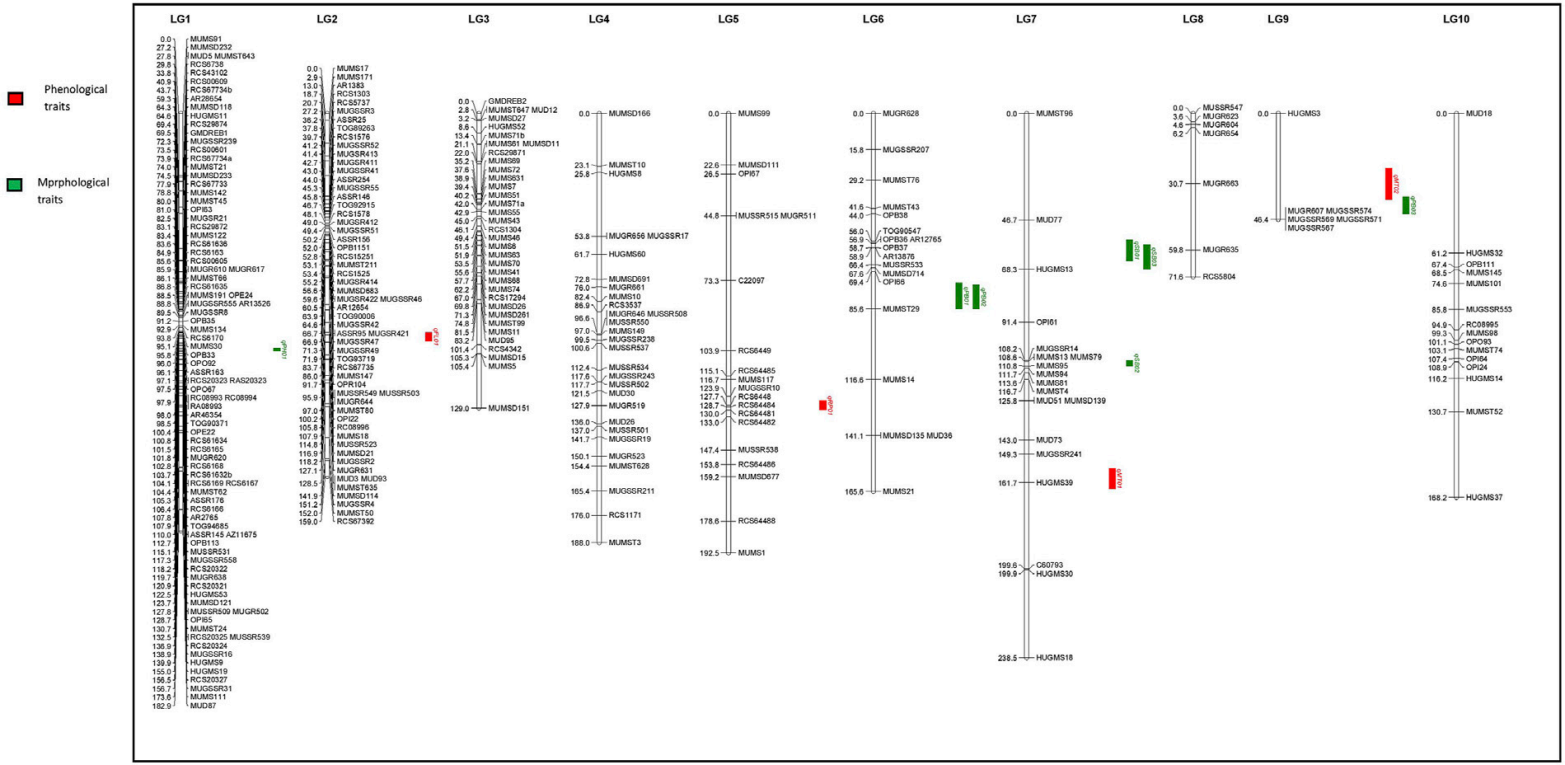
Quantitative trait loci (QTLs) linked to phenological and morphological traits on 10 linkage groups of horsegram (Red color indicates QTLs for Phenological traits; Green color indicates QTLs for Morphological traits.

Using JoinMap software, version 4.0, of the total 493 polymorphic markers, 295 (59.84%) were mapped on 10 LGs at LOD 3.5. The linkage map spanned 1,541.7 cM (Kosambi cM) length and an average marker interval size of 5.20 cM (Table 5). These 295 mapped markers include 15 EST SSRs, 87 genomic SSRs, 87 genic SSRs, 22 RAPDs, 73 SSRs from other species, four drought-specific markers, and seven COS markers. Each of the ten linkage groups differed from one another in terms of their length and the total number of markers mapped. Of the total 295 mapped markers, LG1 harbored 89 markers followed by LG2 which contained 58 markers. 35 markers were mapped on LG3, 29 markers were mapped on LG4, 19 were mapped on LG5 and LG7 whereas 18 markers were mapped on LG6. LG eight and LG nine contained the least number of markers with seven and 6 markers, respectively, and linkage group 10 harbored 15 markers (Figure 4 and Table 5). Though LG7 is having a maximum length of 238.5 cM due to very less number of markers 19) present on it with an average marker density of 12.5 however is of less importance while mapping of different QTLs in comparison to linkage groups LG1 and LG2 having marker density of 2.0 and 2.7 respectively even having smaller linkage size (182.9 and 159 cM). Segregation distortion for all the 493 polymorphic markers was determined and of these 295 (59.83%) followed the expected segregation ratio, whereas 198 markers (40.16%) were found to show deviation. These distorted markers were excluded from the final analysis.

**TABLE 5:** Distribution of 295 markers on ten linkage groups of an intra-specific linkage map of horsegram.

LGs	Markers mapped	Map length (cM)	Average marker density (cM)
LG1	89	182.9	2.0
LG2	58	159.0	2.7
LG3	35	129.0	3.7
LG4	29	188.0	6.5
LG5	19	192.5	10.1
LG6	18	165.6	9.2
LG7	19	238.5	12.5
LG8	7	71.6	10.2
LG9	6	46.4	7.7
LG10	15	168.2	11.2
Total	295	1,541.7	5.2

The maximum distance recorded between two markers was 61.2 cM on LG7 and the minimum distance was 0.003 cM on LG2. The number of markers present in different linkage groups was unequal. Four large groups having 12–19 markers within a length of 10 cM and five groups having 28–31 markers within a length of 30 cM were found. The length of the linkage groups did not reflect the number of markers linked on it as the distance between markers varied across different linkage groups. For example, LG1 carrying 89 markers having a length of 182.9 cM and an average marker distance of 2.0 cM, whereas LG4 having a length of 188.0 cM carrying 29 markers and an average marker distance of 6.5 cM, and LG5 having a length of 192.5 cM covered by 19 markers and average spacing of 10.0 cM.

### QTL Mapping

The QTL analysis identified four QTLs (LOD ≥2.5) for phenological and seven QTLs (LOD ≥2.5) for morphological traits (Figure 4 and Table 6). One QTL for days to 50% flowering (qFT01) was detected on LG2 flanked by markers MUGR644-MUMST80 at LOD score of 2.8 and explaining 6.62% of the phenotypic variation with allelic contribution by HPKM249 resulted in reduced flowering time by about >2 days. One QTL for the reproductive period (*qRP01*) was detected on LG5 flanked by MUGSSR10-RCS6448 at a LOD score of 2.7 and explaining 6.36% of the phenotypic variation (Table 6). Two QTLs namely *qMT01* and *qMT02* were detected for days to maturity on LG7 flanked by markers MUGSSR241-HUGMS39 at LOD 2.6 explaining 7.25% of the phenotypic variation and on LG9 flanked by HUGMS3-MUGR607 marker interval at LOD 2.9 explaining 47.53% of the phenotypic variation, respectively and in combination explained 54.78% of total phenotypic variation. Additive effect is the difference in the average performance of the RIL carrying early maturity allele of first parent with respect to those carrying the allele of second parent at the particular locus (QTL). A positive value (+) of the additive effect indicates the allele originating from HPKM249 and a negative value (–) of the additive effect indicates the allele originating from HPK4.

**TABLE 6:** QTLs for various drought related traits identified using QTL Cartographer.

Trait	RIL (HPKM249 × HPK4)		LG	QTL name	Marker interval	LOD score	Additive effect^c^	Pve (*R* ^2^%)^d^
	Year	Loc						
Morphological								
Plant height	2016-2017	COMBINED	1	*qPHT01*	RCS6168-RCS6169	2.7	3.96	6.6
Primary branches	2017	PLP	6	*qPB01*	OPI66-MUMST29	4.2	0.37	22.0
	2017	PLP	9	*qPB03*	HUGMS3-MUGR607	5.4	−0.63	32.4
	2016-2017	COMBINED	6	*qPB02*	OPI66-MUMST29	3.8	0.34	17.0
Secondary branches	2017	BJR	7	*qSB01*	MUD77-HUGMS13	4.9	1.21	23.6
	2017	BJR	7	*qSB02*	MUMS13-MUMS95	3.3	−0.75	7.5
	2016-2017	COMBINED	7	*qSB03*	MUD77-HUGMS13	3.7	0.50	15.5
Phenological								
Days to 50% flowering	2016-2017	COMBINED	2	*qFL01*	MUGR644-MUMST80	2.8	1.19	6.62
Reproductive Period	2017	BJR	5	*qRP01*	MUGSSR10-RCS6448	2.7	3.87	6.36
Days to Maturity	2016	PLP	7	*qMT01*	MUGSSR241-HUGMS39	2.6	2.86	7.25
	2017	PLP	9	*qMT02*	HUGMS3-MUGR607	2.9	7.82	47.53

Additive effect demonstrated that HPKM249 contributed alleles for a reproductive period and days to maturity with QTL named *qMT01* resulted in reduced days to maturity by > 3days and *qMT02* resulted in reduced days to maturity by > 8 days (Table 6).

**FIGURE 5:**
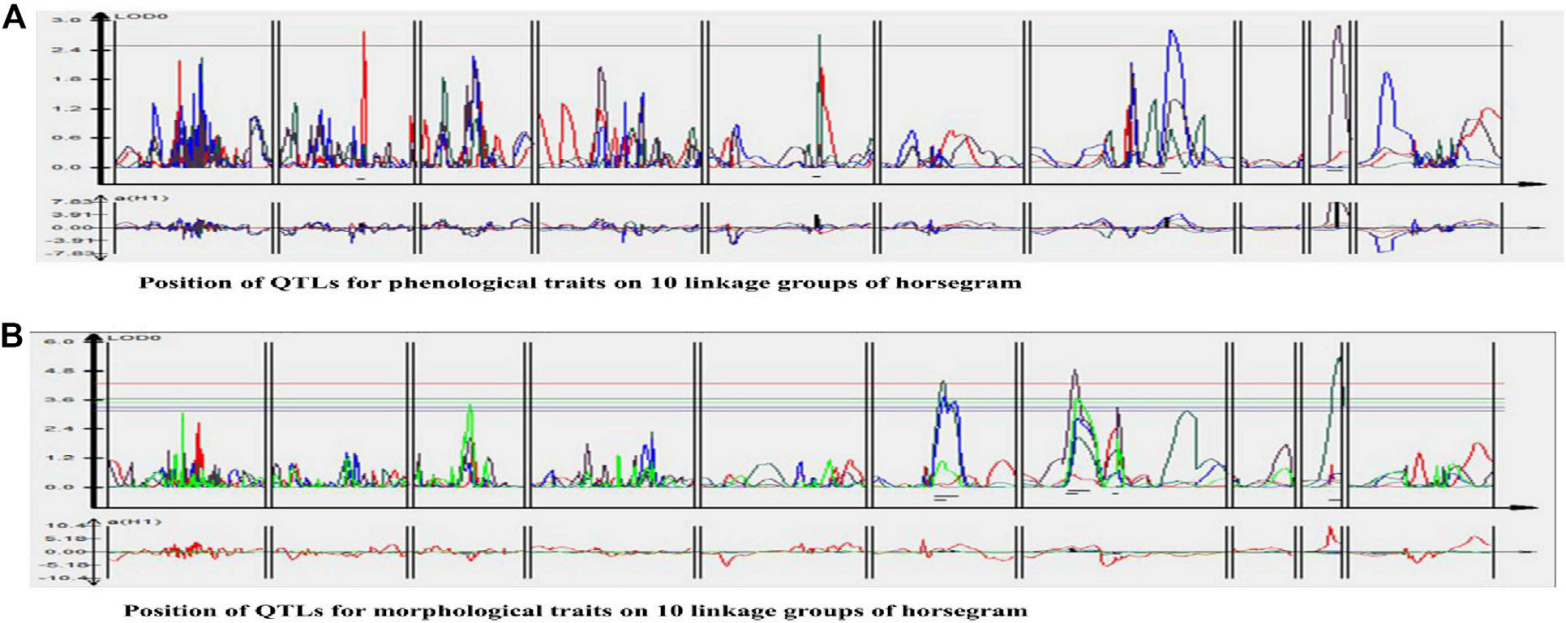
Position of QTLs for **(A)** Phenological traits **(B)** Morphological traits on 10 linkage groups of horsegram.

One QTL was detected for plant height namely *qPH01* on LG1 flanked by RCS6168-RCS6169 and explaining 6.6% of the phenotypic variation at a LOD value of 2.7. This QTL had an additive effect of 3.96 cm and was contributed by the allele from HPKM249. Three QTLs were detected for primary branches namely qPB01, qPB02, and qPB03 with two on LG6 (qPB01 and qPB02) both flanked by OPI66-MUMST29 and explaining 22.0 and 17.0% of the phenotypic variation, respectively, and one QTL on LG9 (qPB03) flanked by HUGMS3-MUGR607 explaining 32.4%of the phenotypic variation (Table 6). These QTLs in combination explained 71.4% of total phenotypic variation for primary branches. Additive effect demonstrated allelic contribution from both the parents. Three QTLs were detected for secondary branches all on LG7 namely *qSB01, qSB02,* and *qSB03*. Both *qSB01* and *qSB03* were flanked by MUD77-HUGMS13 marker interval explaining 23.6 and 15.5% phenotypic variation respectively. *qSB02* was flanked by MUMS13-MUMS95 marker interval explaining 7.5% of phenotypic variation. These QTLs in combination explained 46.6% of total phenotypic variation for secondary branches. Additive effect demonstrated allelic contribution from both the parents. The position of QTLs for phenological and morphological traits at LOD ≥2.5 on 10 linkage groups on horsegram has also been shown in Figure 5.

## Discussion

The main concern of the breeders and farmers is to enhance the yield of the important crops which can provide sufficient nutrition to the human population. Genetic improvement of underutilized crops and including them in commercial agriculture for more production to address this issue. Horsegram is an important legume with great potential due to its medicinal, nutraceutical and capable in growing harsh environmental advantages but is still underutilized and understudied. It serves as an important source of protein for the poorest of poor society. It is grown as food by local people in some areas of developing countries and as feed for animals in dry areas (Chahota et al., 2013; Fuller and Murphy, 2018). However, its genetic improvement is ignored by both at scientists as well as at institutional levels. For genetic improvement of any crop, genetic information of its important traits is required. Phenological and morphological traits play an important role in enhancing productivity, adaptation and yield stability of any crop. With the current scenario of climate change and increase in feeding population the need to develop high yielding, early maturing and climate resilient varieties has increased. Different horsegram varieties exhibit variation in their flowering and maturity time therefore genetic knowledge of their phenology aids in the development of varieties with desired and useful characteristics such as early maturing and with high yield. Days to flowering has a direct implementation on other phenological traits like time to podding and maturity (Gaur et al., 2015) and to enhance the yield, balance between flowering time and maturity is essential (Kong et al., 2018). These traits are quantitatively inherited and are influenced by the environment (Sari et al., 2021). Thus knowing genetics and environmental interactions can help to explicate the intrinsic process of flowering time and maturity. Similarly improving morphological and plant architectural traits is essential for enhancing crop yield. Modulating important plant architectural traits can aid plant breeders in optimizing crop performance and yield (Horton, 2000). Various studies have been reported in legumes explaining the positive association between various morphological traits like plant height, more primary and secondary branches, erect and determinate growth type with improved yield (Jain, 1975; Bahl and Jain, 1977; Sedgley et al., 1990).

Linkage maps and genetic information of a trait are prerequisites for starting any breeding program of desirable crop varieties. Construction of saturated linkage maps using molecular markers will further aid in the localization and mapping of genes/QTLs of different important traits. Since QTLs were first identified in tomato using RFLP-based linkage maps (Paterson et al., 1988), different QTLs for various important traits have been identified in many crops. However, the genetics of important traits in horsegram has not yet been determined and similarly, no QTLs for important traits have been mapped. This may be due to limited variation among cultivars and less available genetic resources in horsegram. In this study, we employed genic and genomic SSRs of horsegram, SSRs from well-characterized legume species and RAPDs to develop a genetic linkage map of horsegram and also identified QTLs linked to phenological and morphological traits. Identification of genomic regions controlling these important traits is the first step towards implementing genomic-assisted breeding in this orphan legume species. Development of genomic resources in horsegram lagged much behind compared to other major pulses. However, in recent years efforts have been made to develop molecular markers through the mining of transcriptome and genomic sequencing data (Sharma et al., 2015; Chahota et al., 2017; Kaldate et al., 2017) which is utilized in the study. Both parents HPK4 and HPKM249 crossed for development of RILs population have a good amount of variation for various phenological and morphological traits (Figure 1 and Table 1). The level of polymorphism in parental line using molecular markers is 24.52% which is comparable to polymorphism observed in other legumes such as 22.1% in chickpea (Radhika et al., 2007), 23.6% in peanut (Hong et al., 2010), 26.8% in adzuki bean (Chaitieng et al., 2006) and 27.02% in soybean (Hwang et al., 2009). The present map is a well saturated intraspecific molecular linkage map of horsegram based on DNA markers containing 10 linkage groups and covering a map distance of 1,541.7 cM (Table 4). Large mapping population size along with better pairing and crossing over of chromosomes of two varieties belonging to the same species between them is the main reason for the development of a saturated linkage map. The length of linkage groups ranged from 46.4 cM in LG9 to 238.5 cM in LG7. The marker density ranged from 2.0 to 12.5 cM, with an average marker density of 5.2 cM showing variation in degrees of saturation of all linkage groups. The variation in saturation of markers on different linkage groups showed that the distribution of markers on each LGs was random. The maximum numbers of markers were mapped on LG1, which harbored 89 markers with the average marker density of 2.0 cM and minimum on LG9 which embraced 6 markers with an average marker density of 7.7 cM (Figure 4 and Table 5). Such discrepancies could probably be eliminated by further saturating the map with more SSRs and SNPs markers (Grisi et al., 2007).

The main bottleneck in the development of high-yielding varieties particularly in grain legumes is the dearth of genomic and genetic information of many important traits. Phenological and morphological traits are considered complex traits exhibiting quantitative inheritance because their phenotypic expression relies on many factors (Kover et al., 2009). The genetic information of the important traits is revealed by dissecting the potential genomic regions harboring QTLs controlling these traits. Also, the breeding improvement of the crops is accelerated through marker-assisted selection (MAS) which is aided by the identification of linked markers to important QTLs. Thus identification of major QTLs is a very essential step for the improvement of grain legumes (Bocianowski, 2013) and in identifying closely linked markers to the specific trait for marker-assisted selection and positional cloning. The present study allows the identification of important genomic regions linked to phenological and morphological traits in horsegram. Based on this genetic map and marker-trait associations, a total of 11 QTLs for these traits were identified. Four QTLs (LOD ≥2.5) for phenological traits (days to 50% flowering, reproductive period and days to maturity) and seven QTLs (LOD ≥2.5) for morphological traits (plant height, primary branches and secondary branches) were detected at different LGs across different environments (Figure 4 and Table 6). The phenotypic variation explained by QTLs ranged from 6.36 to 47.53%. In this study, we reported QTLs for all traits recorded at different environments for two years at two different locations as well as based on the pooled data of all the environments. To the best of our knowledge, this study is the first comprehensive study that reports QTLs for phenological and morphological traits like days to flowering, maturity, reproductive period, number of branches in horsegram. A precise understanding of horsegram architecture and phenology through validation and utilization of the markers linked to the trait of interest in marker-assisted breeding will help in designing better breeding strategies.

Since horsegram lagged behind other grain legumes in the development of genomic resources, recent efforts for the construction of linkage map and identification of important QTLs aids in genetic upliftment of horsegram by enhancing the availability of molecular markers. To further strengthen the application of these QTLs in horsegram genomic-assisted breeding, a saturation of linkage map with more molecular markers and locating more tightly linked markers to important genomic regions should be pursued. Also, the construction of a second-generation high-density linkage map with the inclusion of SNP markers would increase the resolution of QTLs and provide a better picture of the occurrence of these QTLs for future genetic and genomic studies.

## Data Availability

The datasets presented in this study can be found in online repositories. The names of the repository/repositories and accession number(s) can be found in the article/supplementary material.
